# Beyond the replication-competent HIV reservoir: transcription and translation-competent reservoirs

**DOI:** 10.1186/s12977-018-0392-7

**Published:** 2018-02-02

**Authors:** Amy E. Baxter, Una O’Doherty, Daniel E. Kaufmann

**Affiliations:** 1 CR-CHUM, Université de Montréal, Montréal, QC Canada; 2 Scripps CHAVI-ID, La Jolla, CA USA; 3 Department of Pathology and Laboratory Medicine, Division of Transfusion Medicine and Therapeutic Pathology, University of Pennsylvania, Philadelphia, PA USA

**Keywords:** HIV reservoirs, CD4 T cells, Flow cytometry, RNA flow cytometry, Fluorescence in situ hybridization

## Abstract

Recent years have seen a substantial increase in the number of tools available to monitor and study HIV reservoirs. Here, we discuss recent technological advances that enable an understanding of reservoir dynamics beyond classical assays to measure the frequency of cells containing provirus able to propagate a spreading infection (replication-competent reservoir). Specifically, we focus on the characterization of cellular reservoirs containing proviruses able to transcribe viral mRNAs (so called transcription-competent) and translate viral proteins (translation-competent). We suggest that the study of these alternative reservoirs provides complementary information to classical approaches, crucially at a single-cell level. This enables an in-depth characterization of the cellular reservoir, both following reactivation from latency and, importantly, directly ex vivo at baseline. Furthermore, we propose that the study of cellular reservoirs that may not contain fully replication-competent virus, but are able to produce HIV mRNAs and proteins, is of biological importance. Lastly, we detail some of the key contributions that the study of these transcription and translation-competent reservoirs has made thus far to investigations into HIV persistence, and outline where these approaches may take the field next.

## Background

Despite over 30 years of research and the tremendous successes of combined anti-retroviral therapy (ART), HIV remains a chronic disease for which there is no cure. In individuals receiving ART, the amount of circulating virus in the plasma is brought down to undetectable levels, as measured by current standard clinical assays. However, the virus is able to persist in the form of integrated proviruses in a predominantly CD4 T cell reservoir and will rebound from this cellular reservoir if therapy is discontinued [1–5]. Therefore, a key challenge for the field is how to identify cellular reservoirs of HIV [6], and crucially, how to measure the impact of potential cure strategies on the replication-competent reservoir [7] as well as defective proviruses capable of expressing HIV proteins [8, 9].

Multiple techniques have been proposed, developed, and successfully utilized to identify the reservoir. Many of these techniques will be discussed in detail elsewhere in this series. Broadly, the majority of approaches focus on either the very early (DNA), or the very late (infectious virus) products of the viral life cycle. This focus has many advantages, but there are key limitations to be considered. For example, common PCR based techniques including the measure of total and integrated HIV DNA [2, 10] vastly overestimate the size of the reservoir due to the high prevalence of integrated, but “defective” proviruses [9, 11, 12]. On the other end of the scale, the Quantitative Viral Outgrowth Assay (Q-VOA), [4, 5, 13] and variants [14–16] may underestimate the size of the reservoir, as not all replication-competent proviruses are inducible with one round of stimulation [11] or able to propagate in the in vitro conditions required for detection. Crucially, such approaches provide population-level, rather than single-cell level, information allowing only a quantification of the relative size of the reservoir, rather than in-depth reservoir characterization.

**Fig. 1:**
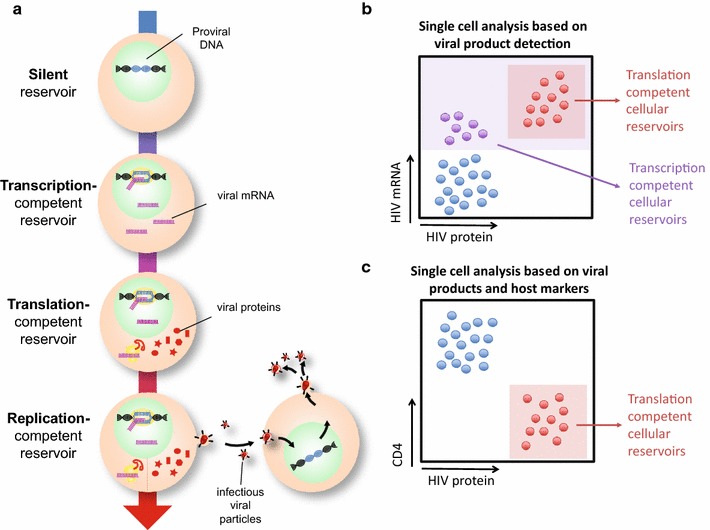
Defining and identifying HIV reservoirs. **a** Schematic detailing the naming conventions used to identify different aspects of the HIV reservoir. **b** Single-cell identification of transcription and translation-competent reservoirs by flow cytometry. **c** Single-cell identification of translation-competent reservoirs, incorporating measures of viral function

With these challenges in mind, we and others have sought a different way of characterizing and understanding HIV persistence (see Fig. 1). For example, while the maintenance of intact, replication-competent viruses is clearly a major barrier to HIV eradication, can transcription or translation-competent proviruses contribute to HIV pathogenesis on ART, and provide key insights into HIV persistence? We suggest that proviruses that may not be fully replication-competent, but that are capable of transcribing viral mRNAs and translating viral proteins, provide an additional dimension to persistence studies; and that the elimination of such proviruses should be considered in the context of a cure. Furthermore, we propose that the in-depth analysis of the cellular HIV reservoir at baseline, i.e. those cells containing proviruses that spontaneously produce viral products in ART-treated individuals in the absence of stimulation or reactivation, enables a deeper understanding and informative quantification of the response to latency reversing agents (LRAs) in the context of “shock/kick and kill” [17] and alternative cure strategies [18–20]. Here, we detail the initial studies of the transcription and translation-competent reservoirs, which have recently overcome issues of specificity and sensitivity, to begin to address these questions.

The approaches we describe uniquely investigate HIV reservoirs at the single-cell level; termed here cellular HIV reservoirs. The use of the word “cellular” distinguishes these measures from the more prevalent population-level analyses utilized in the field. Population-level analysis provide crucial insight into the size and nature of the reservoir; however we and others have demonstrated that studying the reservoir at a single-cell level can provide an additional critical understanding of the heterogeneity of the reservoir.

Lastly, we have avoided the term “latent” when describing these cellular HIV reservoirs since this phrase is commonly used to describe cells containing a provirus that is transcriptionally silent. However, we and others have shown that a rare subset of HIV-infected cells in individuals on long-term ART can express HIV mRNA and proteins in the absence of a spreading infection. By this definition, these cells are not latent at the time of detection, but, as has been suggested, might cycle back to a latent state and thus contribute to the latent HIV reservoir [21, 22].

## Summary of HIV transcription and translation

The transcription and translation of the HIV genome has been studied in detail in vitro (reviewed in [23]). Briefly, the first fully spliced transcripts encode the HIV accessory proteins Tat and Rev [23, 24]. Tat is an essential regulatory protein for viral replication, which binds the HIV TAR (Trans-Acting Response element) RNA, inducing transcription [23]. In concert, Rev promotes HIV RNA nuclear export by binding the Rev Responsive Element (RRE) present in partially spliced and unspliced RNA [23]. Thus, as Tat and Rev protein levels increase, partially spliced RNAs are exported. In this manner, other accessory proteins, in addition to HIV Envelope (Env), are made. Lastly, unspliced mRNA forms are exported to the cytoplasm such that Gag and Pol are also translated, and viral particles are produced.

In addition, there are multiple levels of post-transcriptional regulation that can impact expression of viral mRNAs and proteins. These include mRNA splicing, RNA processing by microRNAs and nuclear export, as well as control at the translation level [23, 25]. In the context of HIV latency, these points of regulation remain underexplored [21, 26]. However, such post-transcriptional regulation should be taken into consideration when measuring HIV reservoirs based on detection of transcription or translation products. For example, a cell that is able to transcribe HIV mRNAs may not be able to translate HIV proteins, due to control at the post-transcriptional level [27].

While many studies have probed the control of HIV expression in T cell lines and activated T cells, little is known about the control of HIV expression in more quiescent or resting primary T cells. It is clear that activated T cells are much more effective at producing infectious virus than quiescent cells, producing 100-fold more HIV Gag RNA per provirus [28]. Whether HIV gene regulation has unique differences between resting and activated cells requires more investigation both in vitro and in vivo; primary models suggest that while splice products form in resting cells, the levels of fully and partially spliced mRNAs are ~ 100-fold lower than in activated cells [28]. Thus, further work building on the lessons learned from the study of in vitro latency models is required to determine how HIV expression is controlled in vivo [29].

## Measuring transcription-competent cellular reservoirs

Relatively early in the epidemic, prior to the discovery and widespread implementation of potent ART regimens, multiple groups reported the detection of HIV RNA species within CD4 T cells from chronically HIV-infected individuals using PCR-based approaches [30, 31]. The advent of potent ART-induced viral suppression saw the detection of such cell-associated (CA)-RNA applied to the latent HIV reservoir. In the late 2000s, Fischer and colleagues provided a key insight into the significance of this transcription-competent reservoir (Fig. 1a) by monitoring multiple forms of RNA within cells, and measuring the frequency of RNA-expressing cells at limiting dilution in HIV-infected individuals as they began therapy. They observed that HIV CA-RNA measures decayed drastically when compared to HIV DNA measures within the same individual [32], and suggested that ~ 5% of cells containing HIV DNA also expressed HIV RNA in individuals on ART [33]. Importantly, more recent work using a nested PCR approach confirmed that the HIV mRNAs detected predominantly resulted from genuine HIV mRNA transcription, rather than chimeric read-through products transcribed from host promoters [34]. This work clearly demonstrated the relevance of cellular RNA-based measures for investigations in cure strategies, and is discussed in depth elsewhere in this series [35]. As with measures of HIV DNA, most classical CA-RNA measures are based upon modified versions of real-time PCR for various HIV mRNA species [36]. Crucially, therefore, this approach provides population-level information, allowing a quantification of the relative size of the reservoir in HIV-infected individuals, but does not enable an in-depth analysis of the cellular nature of the reservoir. With this in mind, we and others have applied various approaches to detect single cells containing a provirus able to produce HIV RNA species; termed the transcription-competent cellular reservoir.

The first studies of transcription-competent cellular HIV reservoirs were performed in the pre-ART era to investigate key questions regarding HIV pathogenesis. In situ hybridization (ISH) for HIV mRNA was used to identify and describe the persistence of HIV-infected cells in the lymph nodes, in particular in the germinal centers, of HIV-infected infected subjects in the clinically latent stage of disease when plasma viral loads are low [37]. Later, a quantification system was developed to enable the frequencies of these HIV mRNA^+^ cells to be compared between tissues and between samples from different individuals [38]. In more recent years, this technique has been transferred to the study of SIV in non-human primate models and has provided valuable insights into the pathogenesis of and immune response to HIV [39, 40]. While powerful, microscopy-based ISH is limited by its relatively low throughput. In the context of chronic, untreated HIV infection, the prevalence of HIV-infected cells is sufficient to enable detection, but still requires laborious analysis of many sections to obtain robust quantitation. However the frequency of such cells is dramatically reduced in ART-treated individuals. Thus, additional, complementary high-throughput techniques were required to investigate very high number of cells to identify these rare events and characterize the cellular reservoir that persisted in individuals on ART.

The late 1990s saw the advent of a new era in immunology; that of multiparametric flow cytometry. This high-throughput approach was soon applied to the study of cellular HIV sanctuaries in HIV-infected, untreated individuals. Patterson and colleagues pioneered an approach based on reverse-transcriptase (RT)-PCR-based amplification and Fluoresence ISH (FISH) detection of intracellular HIV RNA [41], and later a probe-based approach termed SUSHI (simultaneous ultrasensitive subpopulation staining/hybridization in situ, [42–44]). While these approaches provided a key proof of concept for the field, as the authors note, the frequencies of HIV mRNA^+^ cells detected with these assays are generally higher than would be predicted based on measurements of integrated HIV DNA [41]. This indicates a potential issue with false positive detection that may hamper interpretation of this data.

Building on this pioneering initial work, in recent years a new version of these ISH technologies sought to overcome the issues of high background/nonspecific staining and low signal-to-noise ratios, which limited earlier iterations. In 2012 Wang et al. [45] detailed a microscopy technique known as RNAscope. This approach builds on a branched DNA (bDNA) technique described previously [46], but added additional levels of stringency to reduce off-target binding. Briefly, a series of DNA probes are designed whereby each probe has two sections; the first recognizes the target mRNA and the second forms part of a conserved “tail” sequence. The probes are designed such that pairs of probes which recognize adjacent regions of the target mRNA each contain one half of this conserved tail. Only this combined “tail” sequence can be recognized by a DNA pre-amplifier, which in turn is recognized by a secondary amplifier. This amplified structure is then labeled with a fluorescent probe, or an alkaline phosphatase or horseradish peroxidase (HRP) molecule. The requirement for the two probes (known as a “Z”) to bind adjacent to one another in order for the pre-amplifier to bind substantially reduces off-target binding.

Those in the HIV cure field quickly recognized the significance of this approach. The application of this technique to microscopy has been advanced in particular by the Estes laboratory, who have demonstrated the increased sensitivity and high specificity of this assay when compared to alternative ISH approaches (see Table 1 [47, 48]). The low background is particularly striking; the team imaged nearly 70 mm^2^ of uninfected tissue from rhesus macaques and identified only two false-positive RNA^+^ cells [47]. Recently, this group has successfully applied this technology to quantify transcription-competent cellular SIV reservoirs across a broad range of tissues in both untreated and ART-treated animals, confirming the predominance of lymphoid tissues as a key reservoir [49]. While HIV RNA^+^ cells were identified in untreated subjects, further work is required to determine if such cells can be readily identified in ART-treated individuals.

**Table 1:** Comparison of single-cell approaches to measure the transcription- and translation-competent reservoirs

Cellular reservoir measured	Assay	Assay overview	Advantages	Limitations	Potential applications	Key references
Transcription-competent cellular reservoir	RNAflow cytometry	Detection of cells expressing HIV RNA in suspension by fluorescence in situ hybridisation (FISH) using branched DNA (bDNA) technology	High throughput In depth phenotyping of single cells Highly flexible and adaptable	Background observed in HIV-uninfected individuals Labour intensive (2–3 days protocol) High starting cell number required	LRA screening Reservoir quantification In depth phenotyping of the reservoir Biomarker discovery	[52, 55]
	Simultaneous ultrasensitive subpopulation staining/hybridization in situ (SUSHI)	Detection of cells expressing HIV RNA in suspension by fluorescence in situ hybridisation (FISH)	High throughput	Higher than predicted frequencies of mRNA^+^ cells observed	Reservoir quantification Phenotyping of single cells, including myeloid cells	[41–44]
	Conventional in situ hybridization	Detection of cells expressing HIV RNA in situ using radiolabelled or enzymatic detection	Tissue level information	Limited cell phenotyping Labour intensive	Reservoir quantification in tissues	[37, 38, 47, 48]
	RNAScope	Detection of cells expressing HIV RNA in situ using branched DNA amplification and detection	Tissue level information Highly sensitive and specific Short assay duration	Limited cell phenotyping	Reservoir quantification in tissues/whole body	[47–49]
Translation-competent cellular reservoir	Fiber optic array scanning technology (FAST)	Antibody-based detection of cells expressing HIV protein and down-regulating CD4, in suspension	Relatively high throughput Short assay duration	Specialized microscopy tools and software required Limited cell phenotyping	LRA screening Reservoir quantification	[62]
	RNAflow cytometry	Concurrent detection of cells expressing HIV RNA by FISH using branched DNA (bDNA) technology, and HIV protein in suspension	High linearity and specificity High throughput In depth phenotyping of single cells Highly flexible and adaptable	Labour intensive (2–3 days protocol) High starting cell number required	LRA screening Reservoir quantification In depth phenotyping of the reservoir Biomarker discovery	[53–55]

In parallel, this approach was applied to flow cytometry, and developed by our group and others in collaboration with the company Affymetrix (now part of ThermoFisher) into a commercial RNAflow assay known as PrimeFlow^TM^. It was quickly utilized for the high-throughput, high-sensitivity detection of cellular mRNAs [50]. Thus far, three groups have reportedly applied this RNAflow technology to the flow-cytometric study of transcription-competent HIV reservoirs (Fig. 1b, Table 1), with variations in terms of the specificity of the assay and therefore applicability of the approach to studying samples directly from HIV-infected, and particularly ART-treated, individuals [51]. While Altfeld and colleagues successfully applied the technique to the detection of in vitro HIV-infected cells and cell lines, they reported that the sensitivity of this iteration was unlikely to be sufficient to detect HIV mRNA-expressing cells directly in HIV-infected subjects [52]. Similarly, we noted that the *GagPol* probes used in this study showed relatively high background (in the range of ~ 1000 *GagPol* mRNA false-positive events per million CD4 T cells in HIV-uninfected donors) precluding the detection of the transcription-competent reservoir in our hands [53, 54].

More recently, however, Grau-Expósito et al. [55] reported a high-sensitivity version of the RNAflow assay which used 50 probes sets designed against the *GagPol* region of the conserved HXB2 genome. While the authors also reported false-positive event detection in HIV-uninfected individuals, this was taken into account by subtracting this “false-positive” detection rate from the frequency of events detected in HIV-infected samples. The group concludes that this allows a data normalization and present data suggesting that this is reproducible between experiments. Indeed, this mathematical approach may enable quantification of the transcription-competent reservoir. However, such an approach relies on the relative stability of the “false-positive” population between experiments, and furthermore this “false-positive” population will still effectively contaminate the true positive HIV-infected population. This contamination therefore precludes an in-depth phenotyping analysis of these rare HIV mRNA^+^ cells, particularly in samples from ART-treated individuals where the frequencies of mRNA^+^ cells is close to the limit of detection.

Thus, while this assay shows great promise, the applicability for the detection of transcription-competent cellular reservoirs in samples from treated patients remains unclear. Previous studies using highly-sensitive, limiting dilution RT-PCR demonstrated that low levels of HIV *gag* mRNA could be detected in a subset, only ~ 5%, of HIV DNA-containing cells in subjects on ART [33]. Using a dilution assay, Grau-Expósito et al. demonstrated that the detection of mRNA^+^ cells was linear down to the lowest dilution tested (50 events per million cells). Accordingly, in samples from untreated HIV-infected individuals, the median frequency of mRNA^+^ events detected was above this threshold at ~ 165 per million CD4 T cells. However, unsurprisingly, these events were much rarer in samples from ART-treated individuals (~ 6–20 per million CD4 T cells in the absence of stimulation [55]). Therefore further validation may be required to ensure that this approach is linear down to the ranges required for the robust evaluation of cure therapies.

A further key consideration of such flow-cytometric mRNA-based detection assays is the sensitivity of these approaches in terms of the number of mRNA copies that a cell must express to be detected. To address this question, Baxter et al. performed a confocal microscopy analysis of CD4 T cells from a HIV-negative individual, processed with the HIV^RNA/Gag^ assay. They observed a mean of ~ 7 false-positive *GagPol* mRNA spots per cell; providing a conservative detection limit of ~ 20 *GagPol* mRNA copies per cell (+3 standard deviations, [53]). This limit enabled identification of ~ 94% of *GagPol* mRNA^+^ cells from a HIV-infected individual. Therefore, an HIV-infected cell containing at least 20 copies of HIV mRNA is highly likely to be truly infected (0.15% false positive discovery rate for a Gaussian distribution); however an infected cell with fewer copies of HIV RNA is more likely to be missed. Crucially, the number of spots per cell was closely associated with the total fluorescence intensity of the cell, suggesting this approach enables a relative quantification of mRNA copy number [53].

Importantly, however, this analysis makes the assumption that each “spot” represents one mRNA copy, which may not be accurate. Furthermore, the number of copies required for detection varies according to the number of probe set pairs that bind to each mRNA; thus the selection of probe sets and the heterogeneity of the target mRNA are key variables [54]. In a hypothetical example, consider two samples. In the first sample, the probe sets and the viral mRNA sequence match perfectly, therefore if 50 probe sets are available, 50 probe sets will bind. In the second sample, there is a high degree of sequence mismatches with the original sequence used to design the probes; although 50 probe sets are available, only ten are able to bind the target mRNA. Therefore, for a cell in the second sample to reach the same total fluorescence intensity as a cell in the first sample, five-times as many mRNA copies may be required. While this is an oversimplification, it demonstrates a key point that these assays may “miss” true HIV-infected cells due to sequence heterogeneity. One potential solution is to design individual probes for each patient after sequencing the patient’s virus, but this may be prohibitively costly. Given this point, and those raised above, work in our laboratory and others is ongoing to increase both the specificity and the sensitivity, and therefore the applicability, of these RNAflow assays to the detection of the transcription-competent cellular reservoirs.

## Measuring translation-competent cellular reservoirs

A key consideration in the measurement of transcription-competent reservoirs is that not all of the cells detected as HIV mRNA^+^ contain proviruses able to produce infectious virions, or even HIV protein (Fig. 1a). Indeed, defective and hypermutated RNAs, including those containing APOBEC-mediated G-to-A hypermutations, have been readily detected in HIV-infected individuals [56–58]. Furthermore, given the high prevalence of defective mRNAs detected following latency reversal/reactivation, it has been hypothesized that RNAs containing major mutations may be more susceptible to reactivation and thus more likely to be detected [57]. Therefore, to add a further level of stringency to these approaches, we and others have focused on the identification of the translation-competent cellular reservoir. We suggest that a cell containing a provirus that is capable of HIV protein translation at a high level is more likely to be replication-competent than a provirus detected only as integrated HIV DNA or capable of producing only HIV RNAs. However, previous reports have indicated that a fraction of “defective” proviruses are capable of producing some HIV proteins, particularly *pol* mutants [8, 58, 59]. Thus, while we acknowledge that not all translation-competent proviruses identified are also replication-competent, we propose that the translation-competent cellular reservoir is substantially enriched for replication competence compared to, for example, the integrated HIV DNA reservoir.

The first compelling evidence that HIV reservoirs could be translating HIV proteins came from in vitro models. HIV Gag protein was used as a target, as this protein is expressed at very high levels in HIV-infected cells and each virion incorporates ~ 5000 Gag particles [60]. HIV Gag was detected in a small fraction of resting T cells after direct infection in vitro (Gag^+^, [28]), however this represented only a minority of the cells containing integrated HIV DNA. Whether these Gag^+^ cells were an in vitro artifact or had a counterpart in vivo was unclear until recently [53, 55, 61]. The first evidence that HIV Gag could be expressed in resting CD4^+^ T cells in vivo came from the sorting of resting HIV Gag^+^ non CD4-lineage negative PBMCs from HIV-infected subjects. In the order of ~ 1 Gag^+^ cell per million PBMCs were detected from ART-treated individuals [61]. However, this technique was labor intensive and fraught with false positives. While Gag^+^ cells were enriched for HIV DNA, only 10% of the sorted Gag^+^ cells contained HIV DNA. Thus, this approach provided key evidence that HIV protein expression likely occurred in T cells in ART-treated subjects, but indicated that more sensitive methods were required.

Detection of HIV cellular reservoirs was further advanced by exploiting HIV’s ability to downregulate CD4 as a surrogate marker for cellular reservoirs (Fig. 1c) [53, 55, 62]. A well-known function of Nef, Env and Vpu is the downmodulation of CD4 in activated T cell infection [63–68]. In vitro experiments showed that after direct infection of resting CD4^+^ T cells a subset of cells with integrated HIV DNA were Gag^+^ and negative for surface CD4, suggesting internalization and downregulation of CD4 [62]. Sorted Gag^+^CD4^−^ cells contained HIV proviruses by Alu-gag PCR, proving the presence of Gag was not due to bound virions. Moreover, extensive phenotyping confirmed that these were genuine TCRαβ CD4 T cells with internalized CD4. Mutational analysis showed that Nef and Env, but not Vpu, were required for CD4 internalization, suggesting that if an HIV-infected cell downregulates CD4 it is likely that additional HIV open reading frames (including *env*, *nef*, *tat*, and *rev*) are intact and expressed. Thus, to express Gag and to downregulate CD4, a large fraction of the 3′ and 5′ regions of the HIV genome must be intact.

These in vitro experiments suggested that an approach combining detection of Gag protein expression with CD4 downregulation could be used to identify translation-competent cellular reservoirs. However, sorting strategies, while useful for proof of principle, proved impractical. Thus, the O’Doherty lab introduced a different approach (Table 1, [62]). They exploited a rare cell detection technique used in cancer detection, FAST (Fiber-optic Array Scanning Technology [69–71]), to scan up to 20 million cells adhered to a slide, followed with Automated Digital Microscopy to confirm the cellular phenotype. Applying this rationale and technology enabled imaging of high numbers of PBMCs from ART-treated patients, stained for intracellular CD4 and Gag protein. Indeed, they identified Gag^+^ cells at low frequencies (0.33–2.7 events per million PBMCs), many of which were CD4^−^, or showed punctate internalized CD4 staining. The absence of surface CD4 suggests that indeed, the majority of these cells contain a translation-competent HIV provirus and are distinct from the false-positive Gag^+^ events observed in HIV-uninfected individuals [62]. The key strength of FAST combined with Automated Digital Microscopy is the lower false positive rate compared to classical Gag staining by flow cytometry. While FAST has the potential to be high throughput, the technique is still in early development, the confirmation of positive results by Automated Digital Microscopy is time intensive and this technology is not widely available. Therefore, alternative methods to detect the translation-competent cellular reservoir were required.

## Combining measures of transcription and translation-competent cellular reservoirs

Combining HIV protein detection with HIV RNA detection provided a key breakthrough to overcome the hurdle of false positive signals, using a high-throughput routinely available technology [52, 53]. These approaches utilize the simultaneous detection of HIV *GagPol* mRNA using the RNAflow technique described above [55], along with concurrent intracellular antibody staining for HIV Gag protein [61], (Fig. 1b, Table 1). While Martrus et al. [52] found that the specificity of this dual-staining approach was also insufficient for the analysis of samples from HIV-infected individuals, Baxter et al. [53, 54] were able to identify translation-competent cellular reservoirs in samples from chronic, untreated HIV-infected individuals and, crucially, in ART-treated individuals following in vitro restimulation. This approach was coined as the HIV^RNA/Gag^ assay. As discussed above, key considerations for such assays are the sequence homology between the probes and the target mRNA and the number of probes required. We designed probes against a lab-adapted strain JR-CSF and found that the redundancy afforded by the use of a high number of probe sets (40 total against *gag* and *pol* [53]) was sufficient to overcome the majority of the sequence heterogeneity in primary subject samples. Crucially, the false positive detection rate when protein and mRNA detection was combined was exceptionally low, with only one HIV *GagPol* mRNA^+,^ Gag protein^+^ (HIV^RNA+/Gag+^) event detected in nearly 8 million CD4 T cells from HIV-negative individuals. In comparison, the high false positive rate based on HIV mRNA or protein expression alone masked the detection of translation-competent cellular reservoirs [54]. Furthermore, this iteration was highly linear and specific; bringing these two advances together enabled the detection of 0.5–1 HIV^RNA+/Gag+^ events per million CD4 T cells.

Importantly, the high specificity and flow-cytometric basis of this approach enabled multi-parameter, in-depth phenotyping of the translation-competent cellular HIV reservoir that were not previously possible. For example, consistent with observations made by the O’Doherty laboratory [62], cells identified as HIV^RNA+/Gag+^ strongly downregulated CD4. Moreover, HIV^RNA+/Gag+^ cells were enriched in the circulating T follicular helper cell population [53] and cells expressing inhibitory receptors, consistent with previous reports [72–75]. These examples demonstrate the importance of a low false-positive detection in measuring HIV cellular reservoirs.

Lastly, while Grau-Expósito et al. [55] focused on the transcription-competent cellular reservoir, they also identified a subset of mRNA-expressing cells which expressed viral Gag protein, and thus were also able to identify the translation-competent reservoir as a subpopulation of the transcription-competent cellular reservoir. An area of key further interest is to determine what features (viral or host) may distinguish these two different reservoirs.

Taken together, this work demonstrates that the detection of multiple HIV viral products, or the downstream consequences of these products such as loss of CD4 expression, can overcome the issue of false positive events. Furthermore, we suggest that this multi-faceted approach increases the likelihood that a translation-competent cellular reservoir contains a replication-competent provirus. Nonetheless, careful controls for false positive signals are imperative and additional work is required to determine which fraction of the translation-competent cellular reservoir is truly replication-competent.

## Why measure transcription and translation-competent cellular reservoirs?

### Closing the gap between DNA quantitation and measures of replication-competent virus

A crucial caveat in the measurement of transcription/translation-competent cellular reservoirs is that not all cells detected by these assays may contain a virus able to initiate a spreading infection in vivo: a replication-competent provirus. However, we suggest that the detection of cells containing proviruses able to produce viral mRNA and proteins is biologically and scientifically relevant. Secondly, we propose that the populations of HIV-infected cells detected by these approaches are likely to be highly enriched for replication-competent virus. Thus, measuring the translation-competent cellular reservoir after latency reversal may be an appropriate and informative surrogate for detection of replication-competent proviruses. Optimistically, such approaches may overcome the gap between the overestimation of the reservoir size measured by DNA-centric techniques and the reported underestimation of the reservoir size by the Q-VOA.

To address this second point, both the Buzon and Kaufmann laboratories observed associations with their measures of the cellular reservoir and DNA-based measures, which commonly overestimate the size of the translation-competent reservoir [76]. Baxter et al. also observed a correlation between levels of integrated HIV DNA and the frequency of the translation-competent cellular reservoir in samples from ART-treated individuals following in vitro stimulation with PMA/ionomycin. Interestingly though, DNA measures and the frequency of HIV^RNA+/Gag+^ cells were not associated at baseline. Importantly, the frequency of the cells detected as transcription/translation-competent cellular reservoirs is substantially lower than the number of copies of HIV DNA detected (~ 160-fold lower [55] and ~ 200-fold lower [53]). This difference suggests that measurement of the transcription/translation-competent cellular reservoirs identify a population that is substantially closer to the replication-competent reservoir than DNA measures.

On the other end of the scale, both groups compared their measures to the Q-VOA, which estimates the replication-competent reservoir at frequencies ~ 1000-fold lower than DNA-based approaches at ~ 1 event per million resting CD4 T cells [76], although this likely represents an underestimation [6]. Interestingly, neither group identified a correlation between the frequency of the transcription/translation-competent reservoirs with the Q-VOA. Crucially, the frequency of events detected were higher than, but in the same order of magnitude as, the IUPM. For example, Baxter et al. [53] identified a median frequency of ~ 4.7 HIV^RNA+/Gag protein+^ events per million CD4 T cells following PMA/ionomycin stimulation, compared to a QVOA reading of 1.4 IUPM (Infectious Units per Million) from the same subjects. The similarities between measurements made by IUPM and the translation-competent reservoir after latency reversal further indicate that these measures close in on the true replication-competent reservoir. There are multiple differences between the assays which could explain the lack of a correlation between these two measurement types, including but not limited to the detection of non-replication competent reservoirs within the transcription/translation-competent cellular reservoir population and the stimulation used [11] and the statistical variation predicted by Poisson distribution when detecting exceptionally rare cells [54]. Such differences should be considered when comparing the two assays.

### Uncovering a unique aspect of the reservoir

A key rationale behind measuring the transcription- and translation-competent reservoirs is the additional level of detailed, complementary information that can be gained from the study of this form of the reservoir. As discussed above, many of the techniques used to identify the transcription- and/or translation-competent reservoirs provide information at a single-cell level, as they are often flow cytometry or microscopy-based. This means that an individual cell can be probed for multiple parameters of interest in addition to HIV RNA/protein, such as cellular activation, exhaustion or memory markers [52, 53, 55, 62, 77]. In contrast, PCR-based techniques and the Q-VOA provide only population-level comparative information (i.e. population A contains a higher proportion of HIV DNA than population B). This is particularly important to consider in the context of the wide heterogeneity of the cellular reservoir; when assessing cure strategies it is of paramount importance to understand how all subpopulations of the cellular reservoir respond, rather than treating the reservoir as a homogenous entity. For example, while it has previously been reported that both the central, transitional and effector memory T cell populations contain HIV DNA, there are conflicting reports regarding whether replication-competent virus is predominantly localized in the central memory compartment [78], or the effector memory compartment [79]. CD4 T cells expressing exhaustion markers including PD-1, LAG-3 and TIGIT have been shown to be enriched for HIV DNA, but this enrichment is further dependent on the state of CD4 T cell differentiation [75]. Furthermore, expression of multiple inhibitory receptors on CD4 T cells prior to ART has been identified as a predictive biomarker of viral rebound following treatment interruption; this suggests that the expression of such markers may also identify a subpopulation of latently infected cells with a higher proclivity to viral transcription [80]. From only these limited examples, it is apparent that analysis of bulk CD4 memory populations would prevent an understanding of these subtleties. While sorting individual CD4 T cell populations for downstream analysis is possible, this becomes less feasible when analyzing exceedingly rare CD4 T cell subpopulations, and quickly limited in terms of the number of populations that can be concurrently analyzed. As the approaches we have described for the analysis of the transcription- and translation-competent cellular reservoirs, particularly those which are flow cytometry-based, overcome these limitations, these techniques will become increasingly useful for in-depth characterization of the HIV reservoir.

An additional strength of these techniques is the ability to compare in vitro models and validation experiments with in vivo-infected T cells. Spina et al. [81] previously indicated the limitations of latency models to fully recapitulate latency reversal, however we suggest that the lessons learnt from in vitro models can advance in vivo research. For example, the in vitro observations of a rare Gag^+^ populations in resting CD4 T cells have been supported by the in vivo detection of this population directly in samples from ART-treated individuals [61, 62]. Using the HIV^RNA/Gag^ assay, the in vitro observation of a downregulation of HLA-Class I on HIV^RNA+/Gag+^ cells was confirmed. In contrast, however, HLA-Class II-expressing CD4 T cells were enriched for both HIV mRNAs and protein only in ex vivo samples [53]. Therefore, such approaches can be used to both investigate HIV biology in vivo, but also to build upon key observations made in in vitro models.

### Quantifying the HIV reservoir at the single-cell level in ART-treated subjects

We further suggest that a highly useful aspect of this type of measurement is the ability to quantify the HIV reservoir in ART-treated individuals at the single-cell level (i.e. directly ex vivo in ART-treated subject samples). Such measurements capture a distinct view of the reservoir; this represents the cells from ART-treated individuals that spontaneously reactivate the provirus to produce HIV mRNA, protein, and perhaps viral particles, in the absence of a spreading infection and/or exogenous stimulation [15, 49, 53]. We speculate that the cells containing transcription/translation-competent virus that are producing HIV mRNA and/or protein might revert to a latent state before dying from viral cytotoxicity or immune clearance [22]. Therefore, investigating these cells could provide insight into the single-cell phenotype of the latent reservoir. In addition, plasma sequences identified during viral rebound following treatment interruption match proviruses in cells that were already expressing HIV mRNA before ART was stopped. This indicates that clones of these proviruses likely contributed to the rebound viremia [56]. Thus, defining those single cells that contain transcription/translation-competent viruses and produce viral products during ART may help identify the cell population from which viral rebound may occur.

Furthermore, we propose that quantification of the cellular reservoir in ART-treated individuals in the absence of stimulation can provide a more nuanced understanding of the reactivation of the latent reservoir in response to stimulation. It should be noted that the persistent HIV reservoir in ART-treated individuals has been extensively studied at the population level. As discussed in detail elsewhere in this review series, classical measurements such as cell-associated RNA and integrated DNA have been used to monitor total HIV reservoir size during suppressive ART [36, 82, 83]. Using these approaches, the reservoir is readily quantifiable. In contrast, the detection of alternatively spliced mRNAs by TILDA did not observe spliced mRNA production without in vitro stimulation in all samples studied [84]. Given these differences, we suggest that the quantification of this persistent reservoir at the single cell level could provide key insights. However, such studies have only been conducted in detail recently. Using single-cell RNAflow based approaches, HIV mRNA-expressing CD4 T cells were robustly identified in samples from 2 of 6 virally suppressed ART-treated individuals [55], while HIV^RNA+/Gag+^ CD4 T cells were detected in 8 samples, from a total of 14 [53]. Using the FAST approach, Gag protein^+^ cells were identified in all five of the subjects studied [61], including one individual who was repeatedly sampled over several years. In those samples where translation/transcription competent cellular reservoirs were detected, the frequencies ranged from ~ 10 mRNA^+^ to ~ 1.0 HIV^RNA+/Gag+^ events per million CD4 T cells. Given these frequencies, we postulate that one of the major issues when monitoring this baseline cellular reservoir is the number of cells studied. The lower the total number of cells analyzed in the assay, the lower the probability of detecting the very rare cells infected with HIV [54]. In studies performed by our laboratories we routinely assess two-four million CD4 T cells [53], or six-eighteen million PBMCs [62] to enable detection of these rare cells. The analysis of such a high number of cells is made possible only by the use of the high-throughput approaches, but nonetheless, detection of these rare cells remains challenging requiring significant expertise and is constrained by the size of available clinical samples. While such limitations must be considered, studying the transcription/translation-competent reservoirs can provide additional information regarding the nature of the HIV reservoir at baseline as well as after stimulation.

### Detailing a biologically relevant population

We suggest here that the transcription/translation-competent cellular reservoir may contribute to both the persistent reservoir and importantly the pathogenesis of HIV on ART, and are thus biologically relevant. If this is the case, these cells, not only cells containing replication-competent viruses, need to be considered in the context of HIV cure.

T cell exhaustion and ongoing immune activation are characteristic features of chronic infections [85], including HIV [86–89], and are driven in part by exposure to persistent antigen [90]. In the presence of suppressive ART, HIV antigen levels should be low, however, p24 and Env protein products can still be detected in the plasma of HIV-infected individuals under long-term (~ 10 years) of suppressive therapy [9]. Furthermore, ultrasensitive techniques have detected very low level viremia in ART-treated individuals [91, 92]. Additionally, the ongoing production of HIV proteins from “defective” proviruses has been demonstrated [8, 59, 62]. Such observations have led to the term “zombie” proviruses, as while “defective” proviruses may not be “alive”, they still may contribute to HIV pathogenesis on ART [59]. These points indicate that the translation-competent cellular reservoir may contribute to continued antigen presence, either through the production of replication-competent virus in the absence of spreading infection at baseline, or through viral protein production only. Crucially though, the precise role of HIV antigen in the persistence of immune activation remains unclear, particularly as HIV antigens are very unlikely to be the only drivers of ongoing immune dysfunction; products from microbial translocation [93, 94] and concurrent viral infections such as CMV and EBV are likely to contribute [95]. While further work is required to determine the significance of the translation-competent reservoir in regards to T cell dysfunction, we suggest that the clearance of such translation-competent cellular reservoirs may need to be considered in addition to the removal of replication-competent virus in the context of a HIV cure.

In addition to contributing to immune activation, viral protein production, possibly from “defective” proviruses may explain the continued presence of antibodies against HIV [9] and indeed may shape the antibody repertoire. Furthermore, a recent study from the Ho/Siliciano lab suggested that cells expressing viral proteins, even from “defective” proviruses, can be recognized and killed by cytotoxic T lymphocytes (CTL) [58]. In support of this finding, other groups have also reported immune-based clearance of HIV-infected cells measured by loss of HIV/SIV DNA, implying that some expression of defective proviruses must be occurring [96–99]. Accordingly, anti-HIV CTL activity in vitro is strongly correlated with viral DNA levels in vivo [61]. As with the antibody repertoire, it is likely that such interactions may also shape the CTL landscape.

## Lessons from the study of transcription/translation-competent reservoirs: an evolving field

The contribution of multiple groups to the study of the transcription/translation-competent reservoirs have provided key insights into the biology of HIV reservoirs, the cellular identity of the reservoir, and the efficiency of cure strategies. A number of groups have reported the association of the size of the transcription/translation-competent reservoirs with subject characteristics and indicators of disease progression in untreated HIV infection. For example the size of these reservoirs is inversely correlated with both CD4/CD8 ratio and plasma viral load [53, 55]. In ART-treated individuals, the CD4 T cell count and CD4/CD8 ratio are important indicators of the immunological response to therapy. A poor reconstitution of the CD4 T cell compartment is associated with increased morbidity and mortality among ART-treated subjects, and is correlated with a larger latent HIV reservoir [100–102]. In line with this suggestion, the level of integrated HIV DNA has been inversely associated with CD4 T cell count [103] and CD4/CD8 ratio [103–106]. Correspondingly an inverse correlation was also observed between the size of the PMA/ionomycin-inducible translation-competent reservoir and the CD4/CD8 ratio [53]. This suggests that a smaller translation-competent reservoir is also associated with increased immunological recovery in response to ART, indicating the potential clinical significance of this reservoir measure.

The approaches pioneered to investigate the HIV cellular reservoir have raised the possibility that there may be distinctions between the subsets of cells captured as the translation-competent versus the transcription competent reservoir. For example, T cell memory populations, in particular the central memory population, contains the majority of HIV DNA in subject on ART [103]. While Baxter et al. [53] observed a comparable distribution between the central and effector memory subsets of HIV^RNA+/Gag+^ cells, Grau-Expósito et al. [55] observed that the effector memory population contained a significantly higher frequency of mRNA^+^ cells than all other memory subsets. Furthermore they identified the same enrichment in the baseline transcription-competent reservoir in ART-treated individuals. While further work is required to determine if the discrepancies between the Buzon and Kaufmann lab studies represent a biologically significant difference between the transcription and translation-competent reservoirs, or if this variation is due to experimental/technical or cohort differences, these data demonstrate the variety and detail of information that such techniques can provide.

The power of this single-cell approach is evident in latency reversal studies, where the RNAflow approach allows simultaneous monitoring of HIV mRNA^+^ cells, and co-expression of HIV Gag protein, in response to stimulation with PMA/ionomycin and clinical LRAs. For example, while romidepsin stimulation resulted in a ~ fourfold increase in the frequencies of mRNA^+^ cells, the majority of this population did not express Gag protein, in contrast to stimulation with PMA/ionomycin which led to a substantial increase in the frequency of dual expressing CD4 T cells [55]. This difference may be explained simply by the time point studied, as the kinetics of latency reversal are likely to differ between LRAs so the mRNA^+^ cells may become positive for Gag protein at a later time point. In support of the former explanation, when the kinetics of latency reversal was monitored in vitro, an mRNA^+^ population rapidly appeared that became Gag protein^+^ over 48 h [52]. Alternatively, the authors suggest that romidepsin may be able to stimulate HIV transcription, but not translation [27], as has previously been observed in vitro using alternative approaches for inducible reservoir measurement [15, 107]. While in a small clinical trial, romidepsin infusions increased plasma HIV-1 RNA levels in 5 of 6 participants, it has not been determined whether this increase in plasma RNA represents true *de novo* production of virus from reactivated latent proviruses [108], as 3 of these subjects were receiving protease inhibitors as part of their ART. Therefore, further work is required to determine the effectiveness of romidepsin as an LRA.

In complementary experiments, Baxter et al. took a different approach and used this technique to address the question: which subsets of CD4 T cells respond to LRAs in vitro by producing HIV mRNA and protein? Cells were stimulated in vitro with the PKC agonists bryostatin or ingenol [109, 110], and the LRA-responsive cells were phenotyped using the memory markers CD27 and CD45RA. Surprisingly, reactivation of HIV RNA and protein expression in response to bryostatin predominantly occurred in the effector memory compartment, despite the central memory population containing high levels of integrated HIV DNA. Curiously, the same polarization was not seen with ingenol, which induced reactivation in all memory compartments [53]. This initial data suggests, critically, that not all populations of HIV-infected CD4 T cells will respond to all LRAs equally. Although further work is required to validate and expand on this results, this supports the requirement for combination therapies to target the entire latent reservoir and again highlights the importance of considering the single-cell heterogeneity of the reservoir in cure strategies.

## Future perspectives

The studies presented here demonstrate the power of studying the transcription/translation-competent cellular reservoirs. While these insights are tremendously valuable for the cure field, the level of heterogeneity detected thus far has been considerable. With this in mind, many groups have sought to discover a single marker that can be used to identify and robustly discriminate cells containing replication-competent proviruses. For example, CD32a has recently been identified as a promising biomarker for latently-infected CD4 T cells [111]. Therefore, an immediate question is whether the transcription/translation-competent cellular reservoirs are also enriched for this marker; the first published study to do so observed limited enrichment [55]. However, the ability to analyze expression and co-expression of multiple markers at a single cell level means that the techniques used for the identification of the transcription/translation-competent reservoirs can be utilized for screening approaches. This type of analysis has clear potential for use in the identification of biomarkers for latent HIV-infected cells, which could then be preferentially targeted by cure strategies.

The application of such single-cell measurements to clinical cure research is a key next step in the development of these approaches. For example, this approach has the power to determine whether a particular treatment is effective at clearing latent virus from a specific cellular compartment. It remains to be determined, however, how the size of the transcription/translation-competent reservoir may be associated with positive treatment outcome; specifically, if a reduction in the size of the transcription/translation-competent reservoir is associated with a longer time to rebound, or post-treatment control, following analytic treatment interruption. In line with this, it will be important to determine if the detection of the transcription/translation-competent reservoirs can provide useful information, when compared to classical measures of HIV DNA or RNA at a population level in this context.

While most of the work shown here has focused on CD4 T cell as the predominant reservoir, alternative cell populations, such as macrophages, have been shown to be infected with HIV. The contribution of this population to HIV persistence however remains controversial [112–114]. Interestingly, Jambo et al. [115] were able to use a flow-based FISH approach to identify HIV-infected alveolar macrophages in bronchial lavages from chronically infected individuals. While additional studies are required to confirm these results, this initial study indicates the power of such approaches to study cell populations other than CD4 and opening up the number of questions that can be addressed.

Lastly, in this review we have focused on assays using flow cytometry and microscopy as a readout. However, the field is now moving beyond viral mRNA/protein detection by flow cytometry, for example by combining single cell sorting by FACS with detection of multiple SIV mRNAs (including *tat/rev*, *env*, *gag* and *LTR*) by ultrasensitive PCR. While this initial study enabled detailed in-depth profiling of HIV infected cells in SIV infected macaques during chronic untreated infection [116], it demonstrated a large amount of variation both between infected, mRNA+ cells and also between tissues. Furthermore, a recent report has demonstrated the concurrent detection of spliced and unspliced RNA, nuclear DNA and Gag protein by microscopy, using an approach known as multiplex immunofluorescent cell-based detection of DNA, RNA and Protein (MICDDRP, [117]). While the latter study focused on in vitro infection, future work will determine how both of these approaches can be applied to detect HIV-infected cells in ART-treated individuals.

## Conclusions

We propose that the detection of the transcription and translation-competent cellular reservoirs provides a unique, complementary, approach to identify and probe the cells contributing to HIV persistence at a single cell level. While not all cells identified as transcription and translation-competent cellular reservoirs will harbor replication-competent virus, we propose that such cells, particularly those which express multiple HIV mRNAs, express HIV protein and downregulate CD4, are likely to be enriched for replication-competent virus. We base this speculation on the requirement for the functionality of multiple genes to bring about this phenotype, including *gag*, *tat*, *rev*, *env*, and *nef*. Thus, we suggest that these approaches close the gap between alternative reservoir measurements and provide a closer estimate of HIV reservoir size. Finally, we summarize recent evidence supporting the concept that even if such transcription/translation-competent proviruses are not replication-competent, understanding and/or removing this cellular reservoir will be important for the development of cure strategies.

## Abbreviations

ART: anti-retroviral therapy
bDNA: branched DNA
CA-RNA: cell associated-RNA
CTL: cytotoxic T lymphocytes
FAST: fiber-optic array scanning technology
(F)ISH: (fluorescence) in situ hybridization
HRP: horse radish peroxidase
IUPM: infectious units per million
LRA: latency-reversing agent
TAR: trans-acting response element
Q-VOA: quantitative viral outgrowth assay
RT-PCR: reverse transcriptase-PCR
RRE: rev responsive element
SUSHI: simultaneous ultrasensitive subpopulation staining/hybridization in situ
